# Using a Loneliness Measure to Screen for Risk of Mental Health Problems: A Replication in Two Nationally Representative Cohorts

**DOI:** 10.3390/ijerph19031641

**Published:** 2022-01-31

**Authors:** Timothy Matthews, Bridget T. Bryan, Andrea Danese, Alan J. Meehan, Richie Poulton, Louise Arseneault

**Affiliations:** 1 Social, Genetic and Developmental Psychiatry Centre, Institute of Psychiatry, Psychology and Neuroscience, King’s College London, London SE5 8AF, UK; timothy.matthews@kcl.ac.uk (T.M.); bridget.bryan@kcl.ac.uk (B.T.B.); andrea.danese@kcl.ac.uk (A.D.); alan.meehan@kcl.ac.uk (A.J.M.); 2 National and Specialist CAMHS Trauma and Anxiety Clinic, South London and Maudsley NHS Foundation Trust, London SE5 8AZ, UK; 3 Department of Psychology, University of Otago, Dunedin 9016, New Zealand; richie.poulton@otago.ac.nz

**Keywords:** loneliness, mental health, ROC curve, screening

## Abstract

Background: Loneliness co-occurs alongside many mental health problems and is associated with poorer treatment outcomes. It could therefore be a phenomenon of interest to clinicians as an indicator of generalised risk for psychopathology. The present study tested whether a short measure of loneliness can accurately classify individuals who are at increased risk of common mental health problems. Methods: Data were drawn from two nationally representative cohorts: the age-18 wave of the UK-based Environmental Risk (E-Risk) Longitudinal Twin Study and the age-38 wave of the New Zealand-based Dunedin Multidisciplinary Health and Development Study. In both cohorts, loneliness was assessed using the three-item UCLA Loneliness Scale, plus two stand-alone items about feeling alone and feeling lonely. Outcome measures consisted of diagnoses of depression and anxiety and self-reports of self-harm/suicide attempts, assessed via a structured interview. Results: ROC curve analysis showed that the Loneliness Scale had fair accuracy in classifying individuals meeting criteria for all three outcomes, with a cut-off score of 5 (on a scale from 3 to 9) having the strongest empirical support. Both of the stand-alone items showed modest sensitivity and specificity but were more limited in their flexibility. The findings were replicated across the two cohorts, indicating that they are applicable both to younger and older adults. In addition, the accuracy of the loneliness scale in detecting mental health problems was comparable to a measure of poor sleep quality, a phenomenon which is often included in screening tools for depression and anxiety. Conclusions: These findings indicate that a loneliness measure could have utility in mental health screening contexts, as well as in research.

## 1. Introduction

Loneliness has been a growing public health issue in recent years, a trend that has been compounded by the social restrictions imposed around the world in response to the COVID-19 pandemic [1]. Though distressing, loneliness itself is not considered a mental health problem. It is, however, associated with an increased risk for symptoms of psychiatric disorders such as depression and anxiety, as well as self-harm, suicidality and use of mental health services in the population [2]. Moreover, loneliness is associated with poorer clinical outcomes among groups of people who receive treatment for mental health problems [3]. Loneliness may also be of interest to clinicians as a valuable individual risk indicator for psychopathology, as it is analogous to sleep problems or difficulties with concentration, which are present in a number of disorders and are included in widely used screening checklists such as the Patient Health Questionnaire and the Center for Epidemiological Studies Depression Scale [4,5,6]. Though not all individuals who experience loneliness will have co-occurring mental health problems, there is potential scope to utilise a loneliness measure alongside other information as part of a screening tool [7].

Like many psychological constructs, loneliness is often measured using scales consisting of multiple self-report items, with the summed scores reflecting a continuum along which people vary in the population, e.g., [8,9]. However, for clinical purposes, it may be useful to discriminate moderate levels of loneliness that do not warrant clinical attention from more severe levels that may signal a risk for mental health disorders. Many people are likely to experience some degree of loneliness at some point in their lives and so long as this experience is not too severe and resolves in due course, the distress associated with it is not necessarily debilitating [10]. Nonetheless, there may be a certain point at which feelings of loneliness could be a greater cause for concern to clinicians, caregivers or educators. Currently, there is no consensus on a threshold at which a person is deemed to be ‘lonely’ versus ‘not lonely’, nor a threshold that demarcates ‘severe’ or clinically relevant levels of loneliness. The validity of any hypothetical cut-off score on a loneliness measure would therefore need to be interrogated statistically, taking into account its sensitivity (ability to correctly identify individuals with mental health problems) and specificity (ability to correctly classify those unaffected).

A further consideration is the choice of measure. In the research literature, measures vary from well-validated scales to single-item indicators, and the distinctions between loneliness and related constructs such as social support are often not well-demarcated [11]. In the UK, the Office for National Statistics (ONS) has issued official guidance for the measurement of loneliness, as part of the government’s national loneliness strategy [12]. Its recommended ‘gold standard’ entails a hybrid measurement approach: first, it recommends the use of the three-item short form of the UCLA Loneliness Scale developed by Hughes and colleagues [13], one of the most well-known and widely-used loneliness measures. Second, the ONS recommends the use of a single item, “how often do you feel lonely?” This individual item is to be used as an adjunct to the UCLA scale but could be used in lieu of the scale when time constraints prevent a more reliable assessment of this construct in large surveys.

The three-item UCLA Loneliness Scale has good internal consistency and correlates strongly with the original 20-item UCLA Scale, from which the items were selected via factor analysis [9,13]. The scale’s brevity and ease of use make it useful for assessing loneliness in large cohort studies, online surveys and phone consultations. For the same reason, it has the potential to be administered as a quick screening tool in settings where clinicians have a short window of time to determine if their patients present a risk for mental health problems. However, it does not have a designated cut-off score, which could be useful to inform clinical decisions. Conspicuously, the items are indirectly worded; that is, they do not explicitly use the word ‘lonely’, in contrast to the direct wording typically used in single-item measures. Instead, respondents are asked how often they feel “that [they] lack companionship”, “left out”, and “isolated from others”. It has been suggested that directly worded items may be prone to biases due to perceived stigmas attached to the concept of loneliness or unreliability due to differences in the way the concept is understood by respondents [14]. While using a single item is convenient, it could be limiting, and its usefulness in screening contexts may require stronger justification than a validated loneliness scale.

The aim of the present study was to examine the potential use of a loneliness measure in screening for risk of mental health disorders, using data from two birth cohorts aged 18 and 38 at the time of assessment. Loneliness is a common problem among adolescents and young adults and co-occurs frequently with mental health problems at this stage of the lifespan [2]; however, it is important to ascertain whether this finding can be replicated in older age groups. First, we aimed to identify a cut-off score on the three-item UCLA Loneliness Scale that could be used to identify a level of loneliness that signals elevated risk for mental health problems. Second, we compared the accuracy of this scale to a measure of sleep problems, another phenomenon that generalises to multiple psychiatric disorders and is included in many screening instruments. Third, we investigate whether using a single, stand-alone item could be an acceptable alternative to a multi-item loneliness scale. The findings could have applications both in population research, to identify subpopulations at risk for poor outcomes associated with loneliness, and also in primary care or education settings, as part of a screening battery.

## 2. Materials and Methods

### 2.1. Participants

Data in this study were drawn from two nationally representative birth cohorts in the United Kingdom (UK) and New Zealand (NZ). Measures of loneliness and mental health at age 18 were examined in the Environmental Risk Longitudinal Twin Study (E-Risk Study), and measures collected at age 38 are from the Dunedin Multidisciplinary Health and Development Study (Dunedin Study).

#### 2.1.1. Age-18 Sample

The E-Risk Study tracks the development of a 1994–1995 birth cohort of 2232 British children [15]. Briefly, the E-Risk sample was constructed in 1999–2000, when 1116 families (93% of those eligible) with same-sex, 5-year-old twins participated in home visit assessments. The sample (49% male) is representative of socioeconomic conditions in Great Britain, as reflected in the families’ distribution on a neighbourhood-level socioeconomic index (ACORN—A Classification of Residential Neighbourhoods, developed by CACI Inc. for commercial use) [16,17]: 25.6% of E-Risk families live in “wealthy achiever” neighbourhoods compared to 25.3% nationwide; 5.3% vs. 11.6% live in “urban prosperity” neighbourhoods; 29.6% vs. 26.9% in “comfortably off” neighbourhoods; 13.4% vs. 13.9% in “moderate means” neighbourhoods; and 26.1% vs. 20.7% in “hard-pressed” neighbourhoods. E-Risk underrepresents “urban prosperity” neighbourhoods because such households are often childless. A total of 90% of the sample is of white ethnicity. Home visits were conducted when participants were aged 5, 7, 10, 12 and most recently, 18 years (93% participation). At age 18, each twin was interviewed by a different interviewer. The Joint South London and Maudsley and the Institute of Psychiatry Research Ethics Committee approved each phase of the study. Parents gave informed consent and twins gave assent between 5–12 years and then informed consent at age 18.

#### 2.1.2. Age-38 Sample

The Dunedin Study is a longitudinal investigation of health and behaviour in a representative birth cohort. Participants (N = 1037; 91% of eligible births; 52% male) were all individuals born between April 1972 and March 1973 in Dunedin, NZ, who were eligible based on residence in the province and who participated in the first assessment at age 3 [18]. The cohort represented the full range of socioeconomic status (SES) in the general population of NZ’s South Island and as adults matched the NZ National Health and Nutrition Survey on key adult health indicators (e.g., body mass index (BMI), smoking, GP visits) and the NZ Census of citizens of the same age on educational attainment [19]. The cohort is primarily white (93%), matching South Island demographics [18]. Assessments were carried out at birth and ages 3, 5, 7, 9, 11, 13, 15, 18, 21, 26, 32, 38 and most recently (completed April 2019) 45 years, when 94.1% (N = 938) of the 997 participants still alive took part. At each assessment, participants were brought to the research unit for interviews and examinations. The relevant ethics committees approved each phase of the study, and informed consent was obtained from all participants.

### 2.2. Measures

Data from the age-18 wave of the E-Risk Study were collected in 2012–2014, and data from the age-38 wave of the Dunedin Study were collected in 2010–2012. In both studies, loneliness was assessed using the 3-item short form of the UCLA Loneliness Scale [13]. The items ask “How often do you feel… (1) That you lack companionship? (2) Left out? (3) Isolated from others?” In addition to the UCLA Scale, participants completed two directly worded items: “How often do you feel alone?”, taken from the full UCLA Loneliness Scale, Version 3 [9], and “How often have you felt lonely in the past month?”, adapted from an item used in the Center for Epidemiological Studies Depression Scale [6]. All items were scored as “hardly ever” (1), “some of the time” (2) and “often” (3). The 3 items from the UCLA Scale were summed to produce a scale from 3 to 9, and the two directly worded items were used separately as stand-alone variables.

Mental health problems were assessed via a structured clinical interview based on the Diagnostic Interview Schedule [20]. In the present study, we focused on diagnoses of major depressive disorder and generalised anxiety disorder, based on criteria in the *Diagnostic and Statistical Manual for Mental Disorders*, 4th Edition (DSM-IV) [21], as well as self-reports of any self-harm or suicide attempts. In the E-Risk Study, the depression and anxiety assessments had a reporting period of the past year, and the assessment of self-harm covered the period from age 12 to 18. In the Dunedin Study, the depression and anxiety assessments also had a past-year reporting period, while self-harm/suicide attempt was a lifetime measure. The prevalence of each measure was very similar across the two samples (Table 1).

Sleep problems during the past month were assessed in both samples using the Pittsburgh Sleep Quality Inventory [22], an instrument developed for use in psychiatric practice and research. The questionnaire assesses seven ‘components’ of sleep: subjective sleep quality, sleep latency, sleep duration, sleep efficiency, sleep disturbance, use of sleep medication, and daytime dysfunction. These are coded based on participants’ self-reported sleep routines, and responses to questions such as “during the past month, how would you rate your sleep quality overall?” The components are coded from 0 to 3 and summed to create a global scale of sleep problems, with higher scores reflecting poorer sleep.

### 2.3. Data Analysis

A series of receiver operating characteristic (ROC) curve analyses were conducted with depression, anxiety and self-harm/suicide attempts as the response variables, and the summed 3-item UCLA Loneliness Scale as the predictor. We conducted these analyses using the pROC package for R [23]. The accuracy of the loneliness scale in discriminating between people with and without mental health disorders is denoted by the area under the curve (AUC) statistic. An AUC of 1 would indicate perfect classification, with no false positives and no false negatives, while an AUC of 0.5 indicates a classification no more accurate than chance. AUCs above 0.9 are generally considered ‘excellent’, those between 0.8 and 0.9 are considered ‘good’, those between 0.7 and 0.8 ‘fair’, between 0.6 and 0.7 ‘poor’ and below 0.6 ‘failed’ [24].

In order to identify a score threshold on the loneliness scale that optimised the trade-off between sensitivity (proportion of ‘true positives’) and specificity (proportion of ‘true negatives’), two statistics were consulted from the ROC curve analysis: Youden’s J, calculated as sensitivity + specificity − 1 [25], and the distance from each candidate threshold to the top-left of the curve plane. After an optimal cut-off score on the loneliness scale was selected, logistic regressions were conducted to determine the sample-wide average increase in risk for depression, anxiety and self-harm/suicide attempts that would be signified by such a score.

A further series of ROC curve analyses was conducted, with the same three outcome variables (depression, anxiety and self-harm/suicide attempts) but with sleep problems substituted for loneliness as the predictor variable. The AUCs for the 3-item UCLA Loneliness scale and the PSQI Sleep Scale were then compared using DeLong’s test [26] to determine whether the measures of loneliness and sleep problems differed significantly in their ability to classify individuals with and without mental health problems.

Finally, the two stand-alone loneliness items were examined in both samples to determine if a single indicator could show acceptable performance in lieu of a multi-item scale, thus reducing testing burden in screening. As these had only two possible ‘cut-offs’ (‘some of the time’ or ‘often’), the sensitivity and specificity were calculated using contingency tables. These were then compared to the corresponding statistics obtained for the loneliness scale.

Complete data on all variables were available for 2033 participants in the age-18 sample (98% of those assessed) and 946 in the age-38 sample (98%). Missing data were handled using listwise deletion.

## 3. Results

### 3.1. Using the 3-Item UCLA Loneliness Scale to Identify Individuals with Mental Health Disorders

**Figure 1:**
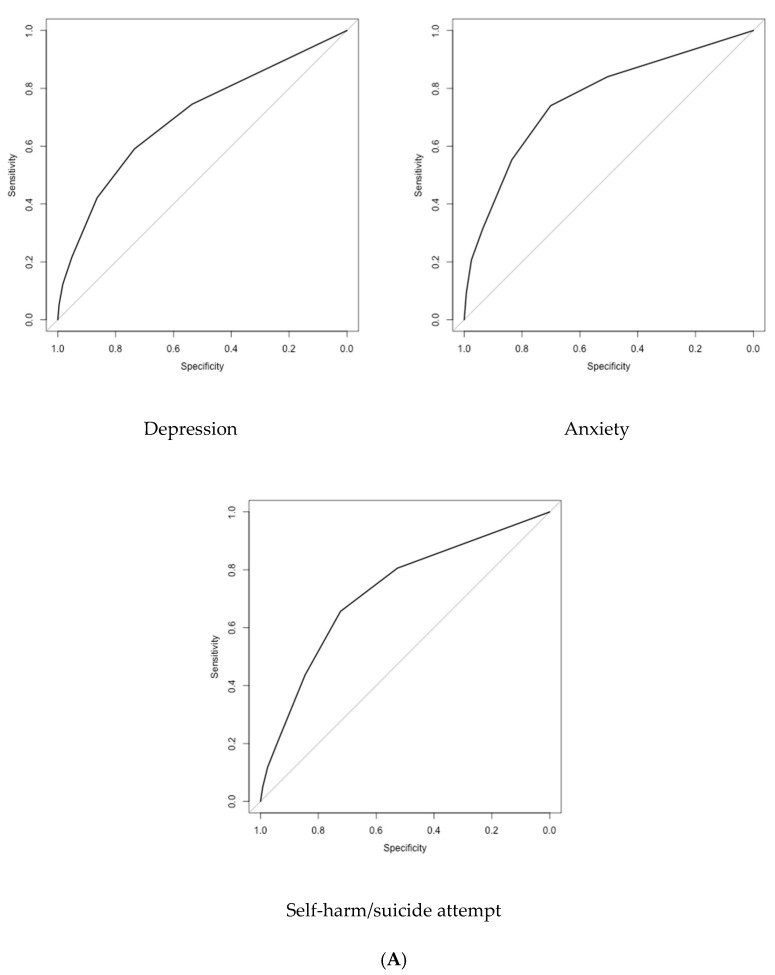

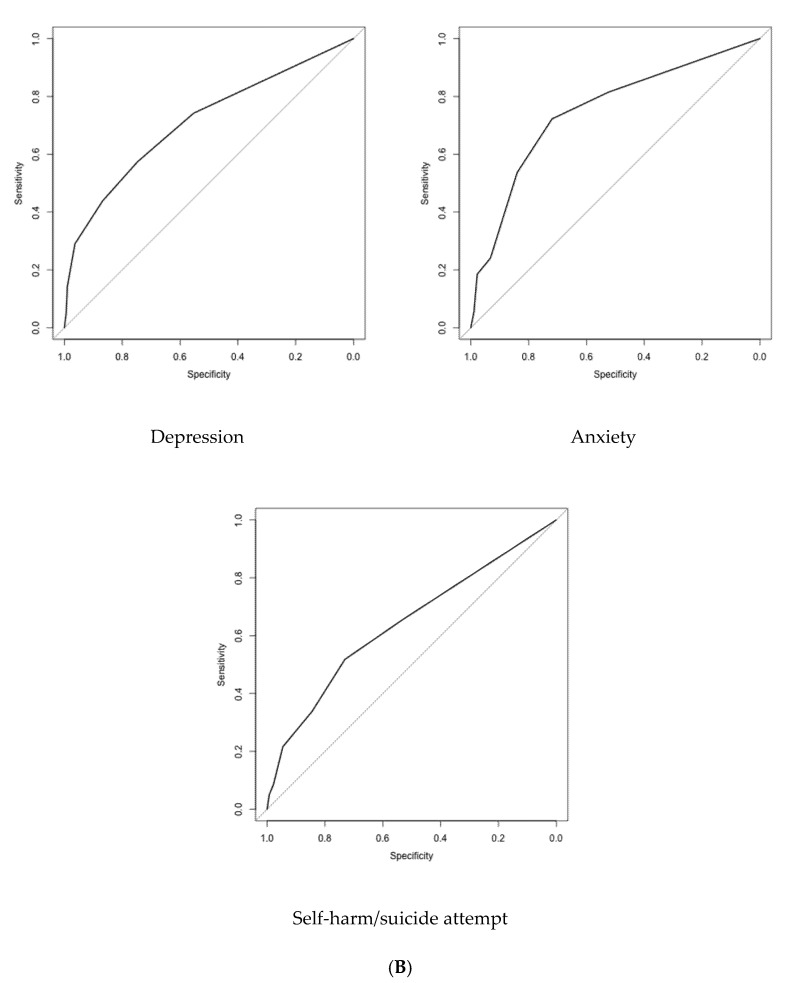
ROC curves plotting the sensitivity and specificity of the 3-item UCLA Loneliness Scale in classifying mental health problems. (**A**) Age-18 sample. (**B**) Age-38 sample.

The ROC curve analyses indicated that the three-item UCLA Loneliness Scale had reasonable accuracy in identifying individuals with mental health disorders. In the age-18 sample, the areas under the curve indicated fair classification of depression, anxiety and self-harm/suicide attempt (Table 2; Figure 1). In the age-38 sample, we found very similar results for depression and anxiety, although the accuracy was poor for self-harm/suicide attempts. When self-harm and suicide attempts were analysed as separate variables, both ROC curves performed similarly in the age-18 sample (self-harm: AUC = 0.72; 95% CI = 0.69–0.76; suicide attempt: AUC = 0.74; 95% CI = 0.69–0.80). However, in the age-38 sample, while the AUC was fair for self-harm (AUC = 0.72, 95% CI = 0.69–0.80), the AUC for suicide attempts was poor (AUC = 0.62; 95% CI = 0.58–0.68).

In both samples, for all three mental health problems, Youden’s J was highest at a score threshold of 5, while the distance from the top-left of the curve plane was lowest at this same threshold. For all three variables, this threshold of 5 showed sensitivity between 0.52 and 0.74, and specificity between 0.70 and 0.75. That is, it correctly identified between half and three-quarters of individuals with a mental health disorder at the cost of a 25–30% false positive rate. Sensitivity was highest for anxiety, and specificity was highest for depression.

**Figure 2:**
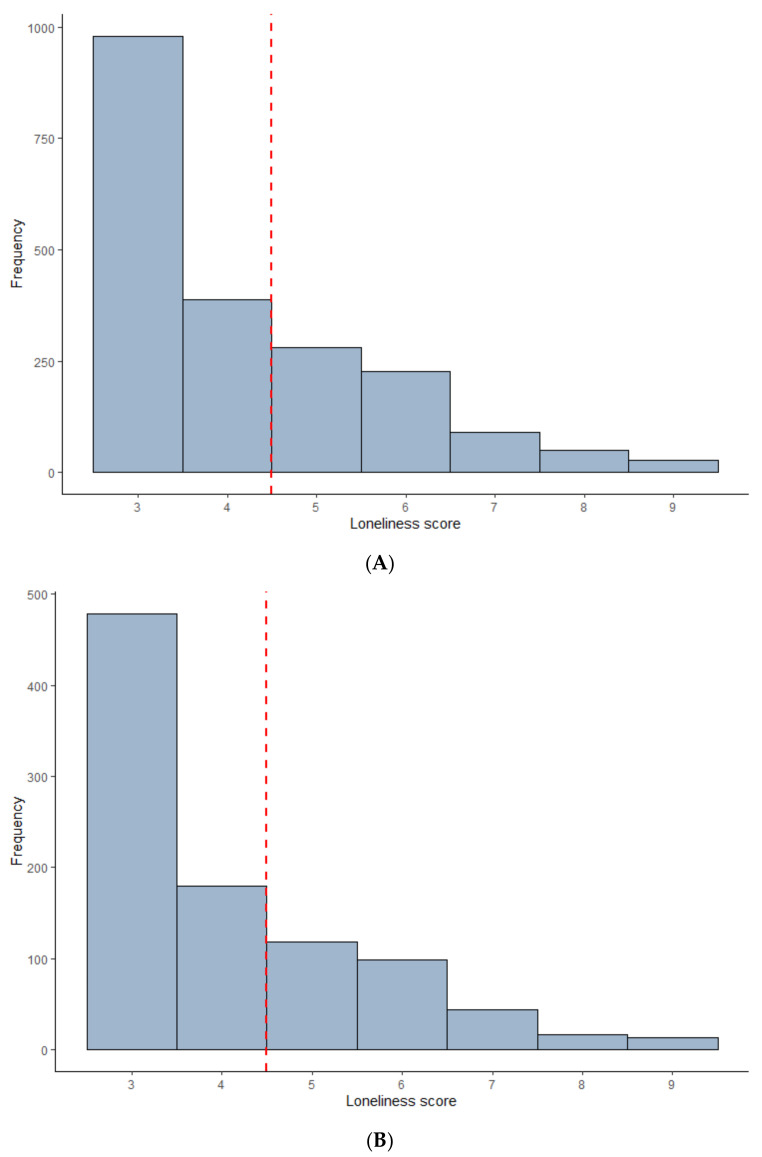
Histograms of 3-item UCLA Loneliness Scale scores, with hypothetical cut-off score of 5 applied. (**A**) Age-18 sample. (**B**) Age-38 sample.

A total of 33% of the age-18 sample and 31% of the age-38 sample scored 5 or above on the Loneliness Scale (Figure 2). To obtain such a score, an individual would have to respond “often” to at least one item or “some of the time” to at least two items. In the age-18 sample, individuals with a score of 5 or higher were four times more likely on average to meet criteria for depression (OR = 4.00; 95% CI = 3.19–5.02), more than six times more likely to meet criteria for anxiety (OR = 6.68; 95% CI = 4.62–9.85) and five times more likely to have self-harmed or attempted suicide (OR = 4.99; 95% CI = 3.84–6.52), in comparison to the rest of the sample. In the age-38 sample, very similar findings were obtained for depression (OR = 3.98; 95% CI = 2.79–5.70) and for anxiety (OR = 6.64; 95% CI = 3.67–12.63). The risk of self-harm or suicide attempt was somewhat lower in the age-38 sample compared to the age-18 sample, though still substantial (OR = 2.92; 95% CI = 2.02–4.22).

### 3.2. Comparing the Accuracy of the 3-Item UCLA Loneliness Scale and a Measure of Sleep Problems

The ROC curve analyses were repeated with the sleep quality scale substituted for the loneliness scale as the predictor. In the age-18 sample, this yielded very similar results to the Loneliness Scale for depression (AUC = 0.71, 95% CI = 0.69–0.74), for anxiety (AUC = 0.71, 95% CI = 0.67–0.75), and for self-harm/suicide attempt (AUC = 0.72, 95% CI = 0.69–0.75). The AUCs for depression and self-harm/suicide attempt did not differ significantly from those obtained using the Loneliness Scale (depression: Z = −0.89, *p* = 0.37; self-harm/suicide attempt: Z = 0.17, *p* = 0.87). The AUC comparison for anxiety was marginally significant (Z = 1.93, *p* = 0.05), indicating that the Loneliness Scale was slightly more accurate in classifying this disorder. In the age-38 sample, the sleep measure had poor accuracy in classifying depression (AUC = 0.61; 95% CI = 0.56–0.66). This was significantly lower than the accuracy of the Loneliness Scale (Z = 2.98; *p* < 0.01). Accuracy was comparable to the Loneliness Scale for anxiety (AUC = 0.70; 95% CI = 0.62–0.77; Z = 1.12; *p* = 0.26) and for self-harm/suicide attempt (AUC = 0.66; 95% CI = 0.61–0.72; Z = −0.59; *p* = 0.56).

### 3.3. Using Individual Loneliness Items to Identify Individuals with Mental Health Disorders

For the individual item “how often do you feel alone?” in the age-18 sample, the response choice “some of the time” had a sensitivity and specificity of 0.60 and 0.75, respectively for depression, 0.70 and 0.71 for anxiety and 0.66 and 0.74 for self-harm/suicide attempt (Table 3). The response choice “often” had a sensitivity and specificity of 0.20 and 0.96, respectively, for depression, 0.25 and 0.95 for anxiety and 0.24 and 0.96 for self-harm/suicide attempt. Similar results were obtained for the age-38 individuals: the option “some of the time” had a sensitivity and specificity of 0.63 and 0.75, respectively, for depression, 0.70 and 0.71 for anxiety and 0.45 and 0.71 for self-harm/suicide attempt. The option “often” had a sensitivity and specificity of 0.17 and 0.98, respectively, for depression, 0.22 and 0.97 for anxiety and 0.11 and 0.97 for self-harm/suicide attempt.

In the age-18 sample, we found very similar results for the item “how often have you felt lonely in the past month?”; the response choice “some of the time” had a sensitivity and specificity of 0.61 and 0.77, respectively, for depression, 0.68 and 0.72 for anxiety and 0.64 and 0.75 for self-harm/suicide attempt (Table 3). The response choice “often” had a sensitivity and specificity of 0.25 and 0.97, respectively, for depression, 0.29 and 0.94 for anxiety and 0.28 and 0.96 for self-harm. In the age-38 sample, however, sensitivity was generally lower and specificity higher for both response choices. “Some of the time” had a sensitivity and specificity of 0.35 and 0.93, respectively, for depression, 0.41 and 0.90 for anxiety and 0.29 and 0.91 for self-harm/suicide attempt. “Often” had sensitivity and specificity of 0.12 and 0.99, respectively, for depression, 0.15 and 0.98 for anxiety and 0.10 and 0.98 for self-harm/suicide attempt.

## 4. Discussion

Whether loneliness is assumed to be an antecedent of mental health problems, an outcome or an epiphenomenon, it serves as a valuable risk indicator that merits the attention of clinicians. This study presents preliminary findings on the ability of a short loneliness measure to discern the presence or absence of common mental health problems. The findings indicate that applying a cut-off score on the three-item UCLA Loneliness Scale could have some value in screening individuals for risk of mental health problems and that a single questionnaire item could be an acceptable substitute (albeit less reliable) where greater brevity is required. The replication of findings in two cohorts indicates that this conclusion holds both for young people and adults.

The accuracy of the three-item UCLA Scale was comparable to that of mental health screening questionnaires of similar length [27]. Statistically, a cut-off score of 5 on the scale (ranging from 3 to 9) was supported by the ROC curve analyses. This is similar to the findings of a recent study among older adults [7], which showed that a cut-off of 3 (on a scale from 0 to 9) using a modified Cantonese translation of the UCLA Scale showed good accuracy at classifying cases of depression. However, the trade-off between sensitivity and specificity can be informed by other priorities besides accuracy alone, such as the cost of committing a false negative versus a false positive [28]. When screening for mental health problems, it could be argued that maximising sensitivity should be the goal, in which case a lower cut-off may be desirable, although this would increase the number of false positives and therefore the potential for over-referral. Nonetheless, we do not propose the use of a loneliness measure as a stand-alone screen for mental health problems but rather as part of a wider battery of items [29]. In research contexts, meanwhile, multiple cut-off scores may be useful for grading levels of severity of loneliness; for instance: ‘mild’ (4–5), ‘moderate’ (6–7) and ‘severe’ (8–9).

The results also indicate that a single item could be an acceptable substitute to the three-item scale in settings where multiple items on a single construct cannot be accommodated. Again, if maximising sensitivity is the goal, then any endorsement of the item (either “some of the time” or “often”) would be appropriate, whereas limiting the threshold to the response choice “often” yielded poor sensitivity. Whether the item was directly or indirectly worded did not appear to make a difference, and the choice of which one to use therefore relies on weighing other considerations. On the one hand, the indirect item belongs to the same parent instrument as the three-item UCLA scale, whereas the direct item is drawn from a depression measure. On the other hand, more people overall endorsed the direct item, suggesting it may benefit from greater face validity. Moreover, the use of a directly worded item is in line with the current ONS guidance [12], the purpose of which is to ensure that loneliness is measured in a consistent manner across different settings. This is particularly important for harmonisation and replication across different studies [30].

### 4.1. Strengths and Limitations

A strength of this study is the replication of the findings in two contemporary, nationally representative cohorts of different ages and nationalities, with close parity of measures. The prevalence and antecedents of loneliness vary across the lifespan, with young adults being particularly affected compared to older adults [10]. The consistency of the findings across both samples is therefore reassuring, as it indicates they generalise to both of these age groups. Moreover, the fact that the findings in a twin cohort were corroborated in a cohort of singletons helps to assuage potential concerns that data on loneliness provided by twins are unrepresentative.

However, there are also limitations to this study. The ROC curve analyses were hampered by the fact that the Loneliness Scale had only three items, with each individual item limited to three response choices. This was intentional, as the aim in this study was to use a short measure that is already in widespread use in large-scale surveys and which could be easily implemented in other research and screening contexts. However, the resulting AUCs were modest, though still substantially greater than 0.5 and within the acceptable range. It bears consideration that these results were obtained using an instrument designed to measure a different construct, rather than one designed specifically to measure depression or anxiety. In view of this, the AUCs are by no means underwhelming and indeed are not unusual compared to other checklists used to screen for psychopathology [24]. The loneliness scale also performed similarly to a measure of poor sleep quality, an impairment that features in many diagnostic and screening tools for mental health problems. There is therefore a plausible case to be made that assessing loneliness as part of the screening process may provide useful added information.

The accuracy of the loneliness scale was generally consistent across all three mental health outcomes in both cohorts, with the exception of self-harm/suicide attempts. While this showed fair accuracy in the age-18 sample, this was not the case in the age-38 sample. There are several possible explanations for this. First, it is likely that this variable captured different behaviours in the two cohorts. In the age-18 sample, the prevalence of self-harm was 14%, versus 4% for suicide attempts. By contrast, in the age-38 sample, only 3% of participants reported self-harm, but 13% reported having attempted suicide. Second, when self-harm and suicide attempts were analysed as separate variables, it appeared to be the latter that was responsible for the low AUC in the age-38 cohort, even though it showed fair accuracy in the age-18 cohort. This is likely due to differences in the reporting periods. The age-18 sample reported on self-harm and suicide attempts within the last 6 years. In the age-38 sample, the reporting period for self-harm was also the past 6 years, but the assessment of suicide attempts covered the entire lifetime. Therefore, some individuals could have been disclosing suicide attempts that occurred many years in the past, introducing error to the ROC curve analysis. More generally, since all of the mental health measures covered a reporting period of at least one year, the results indicate that the Loneliness Scale can classify either current or recent mental health problems. Future research using longitudinal data could further test whether loneliness scores can classify risk for future onset of mental health problems.

Although the study samples were drawn from two different national populations, they were both predominantly of white ethnicity, reflecting the countries’ demographics at the time participants were recruited. Whether the experience of loneliness, its prevalence and its associations with mental health generalise across different ethnicities and cultures is unclear and cannot be inferred from these data. Societal perceptions of loneliness and mental health problems may differ across cultural contexts, and hence the association between these phenomena may similarly vary in strength or be mediated by different processes. The study by Liu and colleagues [7] found similar results to the present study in a sample of older Hong Kong residents; however, further research is merited to attempt replication in other populations. Furthermore, while the findings were replicated in two different age brackets, it is unclear to what extent they hold true in older age, when the experience of loneliness and the reasons for it may differ from earlier stages of the lifespan.

An additional consideration regarding generalisability is the fact that only three outcomes were examined in the present study. These were selected on the basis that they have been previously shown to be strongly associated with loneliness [2]. However, it remains to be established whether these findings are applicable to other mental health problems associated with loneliness, such as psychotic spectrum disorders. Similarly, while the analyses presented here were based on diagnoses according to DSM criteria, the Loneliness Scale may yield different patterns of sensitivity and specificity with other clinical instruments.

### 4.2. Future Research

We suggest that a cut-off of five on the three-item UCLA Loneliness Scale could be viable for identifying clinically meaningful levels of loneliness. However, the real-world utility of this score, versus a slightly higher or lower cut-off, would need to be tested via pilot work in clinical settings. We therefore recommend that these findings be treated as a ‘starting point’. For research purposes, the scale is best used in its original form [13], rather than compromising variation by collapsing it into smaller categories. Nonetheless, a framework for mapping scores onto three or more levels of severity could aid their interpretation in clinical practice.

## 5. Conclusions

Here, we present empirical support for the ability of a loneliness measure to identify, with fair accuracy, individuals meeting diagnostic criteria for common mental health problems, both in young adults and those in mid-life. While loneliness is not a mental health problem in its own right, lonelier individuals are more likely than average to seek help for mental health problems from their GP, a psychiatrist or a counsellor [2]. Loneliness could also exacerbate or impact the prognosis of these co-occurring mental health problems [3]. It is therefore a construct of clinical interest, and the use of a short loneliness measure in screening contexts could thus help to inform clinicians’ decision making.

## Figures and Tables

**Table 1:** Descriptive statistics of loneliness and mental health measures.

	Age-18 Sample (N = 2066)		Age-38 Sample (N = 961)	
Loneliness (Mean/SD)	4.18	1.47	4.11	1.44
Depression (N/%)	414	20%	155	16%
Anxiety (N/%)	153	7%	54	6%
Self-harm/suicide attempt (N/%)	295	14%	150	16%

**Table 2:** Accuracy of the 3-item UCLA Loneliness Scale in classifying mental health problems.

	Threshold	Age-18 Sample					Age-38 Sample				
		Sensitivity	Specificity	PPV	Youden’s J	Distance from Top-Left	Sensitivity	Specificity	PPV	Youden’s J	Distance from Top-Left
	3	1.00	0.00	0.20	0.00	1.00	1.00	0.00	0.16	0.00	1.00
Depression	4	0.75	0.54	0.29	0.28	0.28	0.74	0.55	0.24	0.29	0.27
	5	0.59	0.73	0.36	0.33	0.24	0.57	0.75	0.31	0.32	0.25
	6	0.42	0.86	0.44	0.29	0.35	0.44	0.87	0.39	0.31	0.33
	7	0.22	0.95	0.53	0.17	0.62	0.29	0.96	0.61	0.25	0.50
	8	0.12	0.98	0.64	0.11	0.77	0.14	0.99	0.73	0.13	0.74
	9	0.05	1.00	0.75	0.05	0.90	0.05	0.99	0.62	0.05	0.90
	AUC	0.70 (0.67–0.73)					0.71 (0.67–0.76)				
Anxiety	3	1.00	0.00	0.07	0.00	1.00	1.00	0.00	0.06	0.00	1.00
	4	0.84	0.50	0.12	0.34	0.27	0.81	0.52	0.09	0.34	0.26
	5	0.74	0.70	0.16	0.44	0.16	0.72	0.72	0.13	0.44	0.16
	6	0.55	0.84	0.21	0.39	0.23	0.54	0.84	0.17	0.38	0.24
	7	0.31	0.94	0.28	0.25	0.48	0.24	0.93	0.18	0.17	0.58
	8	0.21	0.98	0.40	0.18	0.63	0.19	0.98	0.33	0.16	0.66
	9	0.09	0.99	0.50	0.09	0.82	0.06	0.99	0.23	0.04	0.89
	AUC	0.76 (0.72–0.80)					0.75 (0.68–0.82)				
Self-harm/suicide attempt	3	1.00	0.00	0.14	0.00	1.00	1.00	0.00	0.15	0.00	1.00
	4	0.81	0.53	0.22	0.33	0.26	0.65	0.53	0.19	0.19	0.34
	5	0.66	0.72	0.28	0.38	0.19	0.52	0.73	0.25	0.25	0.30
	6	0.43	0.85	0.32	0.28	0.34	0.34	0.84	0.27	0.18	0.46
	7	0.21	0.94	0.36	0.15	0.63	0.22	0.95	0.41	0.16	0.62
	8	0.12	0.98	0.44	0.09	0.78	0.09	0.98	0.40	0.06	0.84
	9	0.05	0.99	0.52	0.04	0.91	0.05	0.99	0.54	0.04	0.90
	AUC	0.72 (0.69–0.76)					0.64 (0.59–0.69)				

AUC = Area under the curve. PPV = Positive predictive value. Numbers in brackets reflect 95% confidence intervals.

**Table 3:** Accuracy of individual loneliness items in classifying mental health problems.

**“How often do you feel alone?”**		**Age-18 Sample**		**Age-38 Sample**	
	**Response**	**Sensitivity**	**Specificity**	**Sensitivity**	**Specificity**
Depression	Hardly ever	1.00	0.00	1.00	0.00
	Some of the time	0.60	0.75	0.63	0.75
	Often	0.20	0.96	0.17	0.98
Anxiety	Hardly ever	1.00	0.00	1.00	0.00
	Some of the time	0.70	0.71	0.70	0.71
	Often	0.25	0.95	0.22	0.97
Self-harm/suicide attempt	Hardly ever	1.00	0.00	1.00	0.00
	Some of the time	0.66	0.74	0.45	0.71
	Often	0.24	0.96	0.11	0.97
**“How often have you felt lonely in the past month?”**		**Age-18 sample**		**Age-38 sample**	
	**Response**	**Sensitivity**	**Specificity**	**Sensitivity**	**Specificity**
Depression	Hardly ever	1.00	0.00	1.00	0.00
	Some of the time	0.61	0.77	0.35	0.93
	Often	0.25	0.97	0.12	0.99
Anxiety	Hardly ever	1.00	0.00	1.00	0.00
	Some of the time	0.68	0.72	0.41	0.90
	Often	0.29	0.94	0.15	0.98
Self-harm/suicide attempt	Hardly ever	1.00	0.00	1.00	0.00
	Some of the time	0.64	0.75	0.29	0.91
	Often	0.27	0.96	0.10	0.98

## Data Availability

The dataset reported in the current article is not publicly available due to lack of informed consent and ethical approval, but is available on request by qualified scientists. Requests require a concept paper describing the purpose of data access, ethical approval at the applicant’s institution, and provision for secure data access. We offer secure access on the King’s College campus. All data analysis scripts and results files are available for review.
